# Primary Intracranial DICER-1 Mutant Sarcoma: A Systematic Review of Existing Literature from 2000 to 2024

**DOI:** 10.12669/pjms.41.13(PINS-NNOS).13474

**Published:** 2025-12

**Authors:** Haseeb Mehmood Qadri, Umair Ahmed, Ali Azan Ahmed, Talha Sajid, Ali Hassan, Muhammad Usman Arshad, Pin-Yuan Chen, Asif Bashir

**Affiliations:** 1 Haseeb Mehmood Qadri, MBBS; 2 Umair Ahmed, MBBS.Geisinger Health System, USA; 3 Ali Azan Ahmed, Medical Student Aga Khan University, Karachi, Pakistan; 4 Talha Sajid, MBBS; 5 Ali Hassan, Medical Student Allama Iqbal Medical College, Lahore, Pakistan; 6 Muhammad Usman Arshad, Medical Student Shalamar Medical and Dental College, Lahore, Pakistan; 7 Pin-Yuan Chen, MD, PhD. Department of Neurosurgery, Chang Gung Memorial Hospital, Keelung, Taiwan; 8 Asif Bashir, MBBS, MD, FAANS, FACS. Professor of Neurosurgery, Unit-I,

**Keywords:** Brain Neoplasms, DICER1 protein, Gene Expression Profiling, Human, Sarcoma

## Abstract

**Background and Objective:**

Primary Intracranial DICER1-Mutant Sarcoma (PIDMS) is a rare brain tumor with limited data available on its clinical presentation, treatment, and prognosis. This review aimed to analyze the literature on PIDMS, focusing on its presenting features, imaging findings, genetic profiling, surgical treatment, and outcomes.

**Methodology:**

Our systematic review was conducted following the Preferred Reporting Items for Systematic Review and Meta-Analyses (PRISMA) guidelines. PubMed and Google Scholar were searched to identify the studies published between 2000 and 2024. Only the studies with biopsy-proven PIDMS cases were included. Studies on animals, metastatic, and extracranial sarcomas were excluded. The Joanna Briggs Institute (JBI) critical appraisal tools were used for the quality assessment.

**Results:**

Eight studies comprising 10 patients met the inclusion criteria. Five of the cases (50%) occurred in the pediatric group, while five (50%) in the adult age group (mean age: 15.91 ± 17.71 years). Six patients (60%) were males, and the most common symptoms included headaches and seizures. The frontal and frontoparietal lobes were the most common tumor locations, and the molecular profiling in all 10 cases revealed DICER1 mutation. Gross total resection (GTR) was achieved in 50% of the cases. The mean follow-up duration was 17.5 months, and seven patients underwent combined adjuvant treatment with radiotherapy and chemotherapy. Five patients (50%) had stable disease after undergoing surgical resection and adjuvant therapy.

**Conclusion:**

PIDMS is an aggressive neoplasm with non-specific clinical features overlapping with other brain tumours. Extensive genetic profiling and large-scale clinical trials are needed to develop optimized treatment protocols.

## INTRODUCTION

Sarcomas are malignant tumors of mesenchymal origin, encompassing a heterogeneous group of neoplasms that arise from connective tissues such as bone, cartilage, muscle, and adipose tissue. Among these, spindle cell sarcomas are characterized by elongated, spindle-shaped tumor cells arranged in fascicular or storiform patterns, often posing diagnostic challenges due to overlapping histological features with other malignancies. Primary Intracranial DICER1-Mutant Sarcomas (PIDMS) represent a rare subset, accounting for less than 1% of all central nervous system (CNS) tumors and are thought to originate from multipotent mesenchymal progenitor cells within the leptomeninges or dural layers.^1^,^2^

These tumors predominantly affect children and young adults, with a median age of 19 years at diagnosis, and exhibit a predilection for supratentorial regions, often involving both leptomeningeal and cortical structures.^1^,^2^ Despite its rare occurrence, PIDMS is associated with aggressive clinical behavior, high recurrence rates (up to 28.6%), and poor survival outcomes. It has a median post-diagnosis survival of 3-15 months, or reportedly of six months after the diagnosis of first brain metastasis.^1^,^3^ Ionizing radiation is the only well-established risk factor, though emerging evidence implicates germline DICER1 mutations in a subset of cases, linking PIDMS to hereditary tumor predisposition syndromes.^1^,^4^

The diagnosis of PIDMS relies on histopathological evaluation, supplemented by molecular profiling. Recent studies highlight the utility of immunohistochemical markers such as H3K27me3 loss and TLE1 nuclear expression, which aid in distinguishing PIDMS from mimics like gliosarcoma or meningioma.^1^,^4^ Advanced techniques, including next-generation sequencing (NGS) and DNA methylation profiling, have identified recurrent genomic alterations (e.g., TP53 mutations, MDM2/CDK4 amplifications, RAAS pathway gene mutations, and DICER1 variants), refining diagnostic accuracy and uncovering potential therapeutic targets.^1^ However, significant gaps persist: the majority of data is derived from small, retrospective case series, and standardized treatment protocols are lacking.^2^ A study on the pediatric primary CNS sarcomas from Peru found that there was a very high frequency of association between DICER1 mutation and primary CNS sarcomas, but could not provide any categorical data on the presentation or optimal treatment guidelines for PIDMS.^5^ Current management involves maximal safe surgical resection, radiotherapy, and chemotherapy, yet outcomes remain dismal, with high rates of local recurrence and metastasis.^3^ Limited understanding of molecular pathogenesis and the absence of prospective trials hinder the development of targeted therapies, leaving patients with few effective options.

Despite growing recognition of PIDMS, significant knowledge gaps persist regarding prognostic predictors, optimal surgical margins, and standardized treatment protocols.^1^ This systematic review addresses these deficiencies by pursuing three key objectives: establishing correlation between extent of resection and recurrence patterns, evaluating the prognostic value for comparative treatment efficacy across modalities.^1^,^2^ Our synthesis of global clinical, radiological and molecular data will not only characterize presenting features and refine diagnostic algorithms, but also provide much-needed evidence for developing risk-adapted treatment strategies.^2^ The resulting framework aimed to transform current practice by enabling earlier detection, more accurate prognostication, and personalized therapeutic approaches for this devastating disease.

## METHODOLOGY

**Fig.1:**
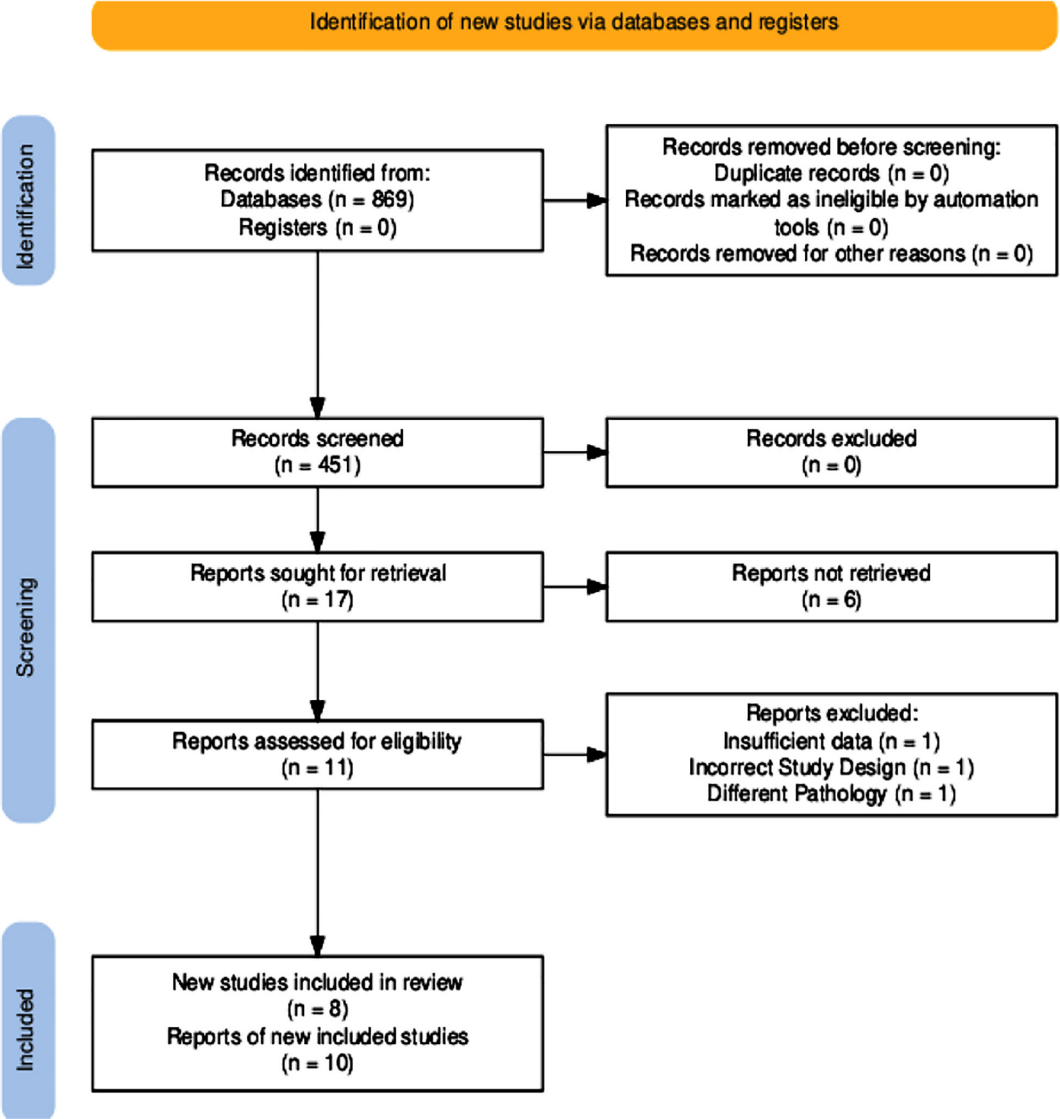
PRISMA Flowchart for the included studies^7^

We conducted our systematic review following the guidelines of Preferred Reporting Items for Systematic Reviews and Meta-Analyses (PRISMA).^6^ The PRISMA flow diagram summarizing the article selection process is presented in Fig.1.^7^ It was registered in the International Prospective Register of Systematic Reviews (PROSPERO) under the registration ID CRD420251001187. The literature search was conducted using two databases: PubMed Central and Google Scholar. These databases were used to find the studies published between January 2000 and December 2024. The search strategy utilized Boolean operators to combine keywords and phrases as follows: (“Primary intracranial” OR “intracranial” OR “brain”) AND (“Sarcoma” OR “sarcomas” OR “spindle cell sarcomas”) AND (“DICER1” OR “DICER1-mutant” OR “DICER1-mutation” OR “DICER1-associated”).

### Inclusion criteria:

- All the relevant case reports, case series, and original articles from 2000-2024, with adequate data available in English language, open access to full text, specifically documenting primary intracranial DICER1-mutant sarcoma.

### Exclusion criteria:

- All the animal studies, cadaveric studies, non-English studies, or those with inadequate or inaccessible data. Additionally, studies conducted on extracranial or metastatic sarcomas were also excluded.

### Data analysis technique:

Four authors extracted relevant data from eligible studies in predefined data collection tables using Microsoft Excel and Microsoft Word. The data included patient demographics, clinical manifestations, imaging findings, hematological profiles, surgical and adjuvant treatments, per-operative and pathological details, and post-operative course. The remaining authors cross-checked and verified the data. Eligible studies were evaluated independently by two authors, and the quality of the studies was assessed using the relevant checklist from Joanna Briggs Institute (JBI) Critical Appraisal Tools, relevant to each study design (Appendix). A summary table of the studies included has been provided in the results section (Table-I)

**Table-I:** Details of demographic data of the study.

Study by	Parameters				
	Study Title	Country of Study	Year of Publication	Gender	Age in years.
Huang et al.^8^	Primary intracranial DICER1-mutant sarcoma: A case report	China	2023	Male	15
Marinelli et al.^9^	A Rare Adult Primary Intracranial Sarcoma, DICER1-Mutant Identified by Epigenomic Profiling: A Case Report	Italy	2023	Male	52
Nejo et al.^10^	Primary Intracranial Spindle Cell Sarcoma, DICER1-Mutant, with MDM2 Amplification Diagnosed on the Basis of Extensive Molecular Profiling	Japan	2022	Female	69
Leelatian et al.^11^	Primary Intracranial Sarcoma, DICER1-Mutant Presenting as a Pineal Region Tumor Mimicking Pineoblastoma: Case Report and Review of the Literature	USA	2022	Female	3
Gigliotti et al.^12^	Malignant spindle cell tumors of the posterior fossa in children: case series and review of management	USA	2021	Male	11
Lee et al.^13^	Primary Intracranial DICER1-Mutant Sarcomas with DICER1 mutation often contain prominent eosinophilic cytoplasmic globules and can occur in the setting of neurofibromatosis type 1	USA, Germany, and Colombia	2020	1: Male 2: Female 3: Male	20 11 19
Rashidi et al.^14^	Teaching NeuroImages: Intracranial DICER1- associated spindle cell sarcoma in a child	United States of America	2020	Male	8
Das et al.^15^	Germline DICER1-mutant intracranial sarcoma with dual chondroid and spindle cell morphology and pulmonary metastases treated with multimodal therapy	India	2019	Female	12

### Ethical consideration:

The study was a review of previously published data, and no direct patient contact was involved.

## RESULTS

The literature search initially yielded a total of 869 articles. After the initial screening, the count came down to 451. Seventeen articles were further screened for eligibility, and full text was retrieved for eleven studies. After full-text screening, a total of six case reports and two case series were included in our study, with 10 patients in total (Fig.1). A summary of the included studies in our systematic review is presented in (Table-I).

The male population was predominant in our study, accounting for 60% (6/10) of the cases, while 40% (4/10) were females. Five patients (50%) belonged to the paediatric age group, while the other five (50%) belonged to the adult age group, with the mean age calculated to be 15.91 ± 17.71 years. The most common clinical manifestations reported in our review were headaches, seizures, and motor weakness occurring in 80% (8/10), 50% (5/10), and 30% (3/10) of the cases, respectively. Nausea, vomiting, cognitive disturbance, sensory deficit, ataxia, photophobia, and phonophobia were reported among other symptoms (Table-II).

**Table-II:** Clinical presentation of the patients where N = 10.

Sr #	Presenting Complaints	Frequency (n)	Percentage Occurrence (n/N) %
1.	Headaches	8	80
2.	Seizures	5	50
3.	Motor Weakness	3	30
4.	Vomiting/Nausea	3	30
5.	Cognitive Disturbance	2	20
6.	Sensory deficit/Ataxia	1	10
7.	Photophobia/Phonophobia	1	10

Magnetic resonance imaging (MRI) was the most implied diagnostic investigation, and the most prevalent sites for sarcomas turned out to be the frontal and frontoparietal regions, occurring in three patients (30%) each. Right-sided lesions were the most common (50%), followed by the left-sided (40%) and central (10%) lesions. Imaging revealed vasogenic edema surrounding the tumour and heterogeneous enhancement with contrast. The details of the radiological findings are provided in (Table-III).

**Table-III:** Details of radiological investigations.

Study by	Parameters			
	T1	T2	Contrast	Others
Huang et al.^8^	Mixed high-low signal intense on T1WI.	Mixed high-low signal intense on T2WI.	Heterogeneous enhancement with a blurred outline to adjacent endocranium.	Edema in the surrounding brain parenchyma and locally presenting high signal on DWI.
Marinelli et al.^9^	Hypointense	Nil	Heterogeneous enhancement with more hypointense component	History of axillary lymphadenopathy due to malignant histiocytosis
Nejo et al.^10^	Hyperintense - enhancing mass lesion at the medial margin of the hematoma	Intraparenchymal Hematoma	No data	Surrounding edema
Leelatian et al.^11^	No data	Hyperintense	No data	Mass showed anterior exophytic extension into the posterior third ventricle, posterior extension into the quadrigeminal cistern, and lateral extension into the left thalamus
Gigliotti et al.^12^	Hemorrhagic posterior fossa mass centered in the dorsal pineal and tectal region with supratentorial extension.	No data	No data	Surrounding vasogenic edema and mass effect on the dorsal midbrain, pons, and fourth ventricle
Lee et al.^13^	Nil	Nil	Nil	Complex, solid and cystic, heterogeneously enhancing masses with meningeal involvement in all three patients; prior hemorrhage, and peritumoral edema
Rashidi et al.^14^	Marked enhancement	Hypointense	Post-contrast enhancement	Area of restricted diffusion, Vascular structures, Remodeling of inner table, hyperdense focus on CT (large vein)
Das et al.^15^	Nil	Nil	Nil	Nil

The extent of surgical resection was documented for nine patients, with gross total resection (GTR) in five (50%), subtotal resection (STR) in two (20%), and near-total resection (NTR) in two (20%) patients. Regarding post-operative complications, two patients had tumour regrowth, while two developed post-operative haemorrhage; one patient with haemorrhage died during the adjuvant therapy. Metastatic disease was not reported in any case. The details of the post-operative course and follow-up are given in (Table-IV).

**Table-IV:** Details of post-operative course and follow-up.

Study by	Extent of Resection	Post-operative complications	Follow-up details
Huang et al.^8^	Gross Total Resection	Nil	Nil
Marinelli et al.^9^	Subtotal Resection	Hemorrhagic episode 2 months post-op.	Patient died while undergoing adjuvant chemotherapy after third neurosurgery
Nejo et al.^10^	Near Total Resection	Tumour regrowth after 2.5 months treated with second surgery	Stable disease after adjuvant radiotherapy and chemotherapy
Leelatian et al.^11^	Near Total Resection	Nil	Decrease in the extent of tumour
Gigliotti et al.^12^	Subtotal Resection	Subdural hematoma extending to the cerebellar tentorium	Stable cranial enhancement without evidence of metastatic disease after adjuvant chemotherapy
Lee et al.^13^	Gross Total Resection in all three cases	Nil	Patient 1# Stable disease with no recurrence Patient 2# Tumour recurrence after four years requiring repeat resection Patient 3# Undergoing adjuvant chemotherapy
Rashidi et al.^14^	Nil	Nil	Nil
Das et al.^15^	Gross Total Resection	Disease progression requiring re-exploration after two weeks of initial treatment	Stable disease with no recurrence after adjuvant treatment; currently on surveillance for DICER-1 syndrome

The most common histopathological findings included the presence of spindle cells, foci of necrosis, hemorrhagic areas, eosinophilic granular intracytoplasmic material, high mitotic activity, pleomorphic nuclei, and high nuclear to cytoplasmic ratio. The histological subtype was specified only by Leelatian et al.^11^ who reported DICER1-mutant sarcoma with rhabdomyosarcoma-like features. Molecular profiling revealed DICER1 mutations in all 10 cases.

The follow-up duration was reported in five studies, ranging from six weeks to four years, with an estimated mean of 17.5 months. Out of the 10 patients, seven underwent combination adjuvant treatment with chemotherapy and radiotherapy. The most common chemotherapeutic drugs were with ifosfamide, carboplatin, vincristine, actinomycin, and cyclophosphamide. There was no data available regarding the adjuvant therapy for the other three patients (Table-V).

**Table-V:** Details of Adjuvant Therapy.

Huang et al.^8^	No Data
Marinelli et al.^9^	***Radiotherapy:***
	The patient received 10 cycles of external-beam radiation therapy (EBRT) at 3 Gray.
	Radiotherapy was interrupted after 9 cycles due to the poor performance status of the patient.
	***Chemotherapy:***
	After the last neurosurgery, the patient received chemotherapy based on doxorubicin and dacarbazine.
	The therapy was interrupted after an unspecified period
Nejo et al.^10^	***Radiotherapy:***
	The patient underwent stereotactic radiotherapy (50 Gy in 10 fractions).
	***Chemotherapy:***
	The patient received 5 cycles of chemotherapy with ifosfamide, carboplatin, and etoposide (ICE).
	***Doses:***
	Ifosfamide: 1800 mg/m² days 1-5
	Carboplatin: 560 mg/m² day 1
	Etoposide: 100 mg/m² days 1-5
Leelatian et al.^11^	No Data.
Gigliotti et al.^12^	***Radiotherapy:***
	Photon-beam radiation therapy (PBRT) using intensity-modulated radiation therapy to the craniospinal axis.
	Total dose: 36 Gy (1.8 Gy in 20 fractions) craniospinal and 14.4 Gy (1.8 Gy in 8 fractions) for the primary site boost.
	Patient received 59.4 Gy to residual tumor, 54 Gy to postoperative bed, 45 Gy to cranial area of leptomeningeal involvement, and 36 Gy to craniospinal axis.
	***Chemotherapy:***
	AEWS1031 (Arm A) protocol:
	Vincristine: 1.5 mg/m² on day 1 only
	Doxorubicin: 37.5 mg/m² on days 1 and 2
	Cyclophosphamide: 1200 mg/m² on day 1 only
	Ifosfamide: 1800 mg/m² and Etoposide: 100 mg/m² on days 1 through 5
	Modified regimen excluded doxorubicin during radiotherapy to avoid radiation recall.
	Consolidation therapy followed by induction phase.
Lee et al.^13^	***Radiotherapy:***
	Cranial radiation following gross total resection.
	***Chemotherapy:***
	Adjuvant chemotherapy with temozolomide.
Rashidi et al.^14^	No Data
Das et al.^15^	***Radiotherapy:***
	Focal radiotherapy (59.4 Gy, 33 fractions) with adequate margins.
	***Chemotherapy:***
	Consensus-driven chemotherapy with vincristine, doxorubicin, and cyclophosphamide.
	Combination chemotherapy including five alternating cycles of VDC/ICE followed by vincristine, actinomycin-D, and cyclophosphamide (VAC).

**Table-I:** APPENDIX Joanna Briggs Institute Checklist for Critical Appraisal of included Case Reports.

Author Name	Q1	Q2	Q3	Q4	Q5	Q6	Q7	Q8	Overall Appraisal
Huang et al.	Y	Y	Y	Y	Y	N	N	Y	Included
Marinelli et al.	Y	Y	Y	Y	Y	Y	Y	Y	Included
Nejo et al..	Y	Y	Y	Y	Y	Y	N	Y	Included
Leelatian et al..	Y	U	Y	Y	Y	N	N	Y	Included
Rashidi et al..	Y	Y	Y	Y	N	N	N	N	Included
Das et al..	Y	Y	Y	Y	Y	Y	N	Y	Included

**Table-II:** Joanna Briggs Institute Checklist for Critical Appraisal of included case series

Author Name	Q1	Q2	Q3	Q4	Q5	Q6	Q7	Q8	Overall Appraisal
Gigliotti et al.	Y	U	Y	Y	Y	Y	Y	Y	Included
Lee et al..	Y	Y	Y	Y	Y	N	N	Y	Included

**Table-III:** Joanna Briggs Institute Checklist for Critical Appraisal of included analytical cross-sectional studies

Author Name	Q1	Q2	Q3	Q4	Q5	Q6	Q7	Q8	Overall Appraisal
Coronado et al.	Y	Y	Y	U	N	N	Y	U	Included

Among the ten cases included in our study, five had stable disease, two had recurrence, while one died while undergoing adjuvant therapy. The final survival outcome was not reported for two patients.

## DISCUSSION

The gaps in understanding the clinical presentation, diagnostic criteria, and treatment of Primary intracranial DICER1-mutant sarcoma (PIDMS) lead to poor patient outcomes globally. Our systematic review summarizes the global evidence, combining clinical, radiological, and molecular information to address these gaps associated with PIDMS patients’ dismal outcomes. Our final review included eight studies from the USA, China, Italy, and Japan between 2019 and 2024. A total of 10 patients with PIDMS were included, consisting of six males and four females; five pediatric and five adult patients. The mean age was 15.91 ± 17.71 years.

PIDMS can occur at any age, and historically, literature has reported a higher incidence among children; however, our review of 10 patients found it to be equally prevalent in the pediatric and adult population.^16^–^18^ According to studies, the location of the tumor and its space-occupying effect determine the clinical symptoms. Cai et al. reported headaches, dizziness, memory loss, and progressive dementia as the primary clinical symptoms.^1^ Headache was the most common presenting symptom in our study as well, reported in 80% of our patients. Our findings expand on the clinical presentation of PIDMS further, reporting other complaints such as seizures, motor weakness, sensory deficit, ataxia, and photophobia/phonophobia.

Existing literature reports tumor laterality to be more prevalent on the right (48.7%), compared to both left (35.9%) and midline (14.5%).^18^ Our study results align with these literature reports, with a 50% occurrence of the disease on the right side. Multiple studies have historically demonstrated greater PIDMS occurrence in the frontal and temporal lobes. Al-Gahtany et al. and several other studies report PIDMS as supratentorial and primarily within the parietal and temporal lobes.^19^–^23^ Haider et al.’s review of 78 patients reported that a majority of primary CNS sarcomas were found in the parietal lobe (17.9%), followed by the frontal (14.1%) and temporal lobes (14.1%).^24^ However, our findings reported the frontal and fronto-parietal lobes as the most frequent tumor locations at 30% each.

Haider et al. further reported that partial resection (57.7%) was preferred over complete resection (42.3%). Finally, the review concluded recurrence in 21 patients (26.9%), with seven recurrences (33.3%) being treated with repeat surgical resection.^24^ A single-center retrospective study conducted by Maher et al. included 13 patients with PIDMS. The study included five patients who underwent gross total resection (GTR), seven with subtotal resection, and one with a biopsy.^25^ These recurrences were found during a second-look surgery in patients who underwent GTR. In addition, two of the largest retrospective studies concerning PIDMS both reported similar findings. Al Gahtany et al.’s study included 16 patients, where nine underwent GTR and seven had a subtotal resection.^19^ Recurrence was found in three of these GTR patients who subsequently had a second surgery. Benesch et al.’s study of 19 patients had 11 patients with GTR, reporting progression of disease in four patients and complete tumor remission in one patient - the highest among all other surgical extent approaches.^26^ The survival outcomes for 70 patients in the study by Coronado et al.^7^ were reported to be significantly better with gross-total resection, 81% (95% CI: 70%–95%) for GTR vs. 25% (95% CI: 8%–73%) for incomplete resection. The combination therapy (surgery + chemotherapy + radiotherapy) were reported to be superior to radiotherapy alone with an overall survival of 71% (95% CI: 57%–87%) with combination treatment compared to 46% (95% CI: 24%–87%) with radiotherapy alone. Our study found gross total resection to be a more successful approach from a curative standpoint. It revealed a better prognosis with the combination of gross total resection and adjuvant therapy, with recurrence occurring in only one of the five cases and no mortality. Patients who had near total resection (NTR) also had stable disease, but one of the patients who had subtotal resection (STR) died while undergoing chemotherapy. This finding endorses the superior curative potential of GTR and NTR, compared to STR, for treating PIDMS (Table-IV). Since the role of adjuvant therapy in intracranial sarcomas remains under-reported in the existing literature, especially in adult patients, our study provides a unique outlook on the efficacy of combination.

### Limitations:

Due to the rarity of PIDMS, the review included only case reports and case series, which are retrospective studies and can have potential selection and publication biases. We tried to minimize these biases by applying strict inclusion criteria. However, they still lack uniform treatment protocols, which limits generalizability of results. Moreover, the study involves a total of only 10 patients, limiting the statistical significance of our results. A greater cohort of patients from a more diverse set of treatment settings could have significantly added to the scientific validity and statistical significance of the review.

## CONCLUSION

Primary intracranial DICER1-mutant sarcoma is a rare brain tumour, predominantly occurring in the pediatric population. It usually involves the frontal or frontoparietal lobes, and the earliest symptoms include headaches and seizures. Our review of ten cases indicates that gross total resection combined with adjuvant therapy improves survival. Neuroimaging and comprehensive genetic testing are essential to guide diagnosis, and early surgical intervention leads to better patient outcomes. Prospective multi-center registries and international collaboration are essential to generate treatment algorithms and improve prognosis.

### Clinical recommendations:

A high degree of suspicion should be maintained for PIDMS, whenever patients, particularly children present with persistent headaches, seizures, or unexplained neurological symptoms. The presence of frontal or fronto-parietal lesions on neuroimaging should prompt early biopsy and molecular profiling to help establish an early diagnosis and guide treatment planning. Adjuvant therapy with chemotherapy (with regimens such as ICE or VAC) and radiotherapy must be included in the treatment plan to reduce the risk of recurrence. A multidisciplinary approach involving specialties including neurosurgery, neurology, oncology, pathology, and genetics is essential to ensure optimized treatment plans and survival outcomes for the patients.

### Author`s Contribution:

**HMQ:** Concept and design of the study, analyzed and interpreted the data, and critically reviewed the manuscript.

**UA:** Analyzed and interpreted the data, manuscript drafting and critical review.

**AAA, TS, AH and MUA:** Data acquisition and interpretation and manuscript drafting.

**PYC, AB:** Data interpretation, critical review of the manuscript and supervision.

All authors have read and approved the final version of the manuscript to be published and are accountable for the integrity of the study.
